# Can Public Health Risk Assessment Using Risk Matrices Be Misleading?

**DOI:** 10.3390/ijerph120809575

**Published:** 2015-08-14

**Authors:** Shabnam Vatanpour, Steve E. Hrudey, Irina Dinu

**Affiliations:** 1 School of Public Health, University of Alberta, Edmonton, AB T6G 1C9, Canada; E-Mails: vatanpour@ualberta.ca (S.V.); idinu@ualberta.ca (I.D.); 2 Faculty of Medicine & Dentistry, Division of Analytical and Environmental Toxicology, University of Alberta, Edmonton, AB T6G 2G3, Canada

**Keywords:** risk matrix, risk priorities, decision-making

## Abstract

The risk assessment matrix is a widely accepted, semi-quantitative tool for assessing risks, and setting priorities in risk management. Although the method can be useful to promote discussion to distinguish high risks from low risks, a published critique described a problem when the frequency and severity of risks are negatively correlated. A theoretical analysis showed that risk predictions could be misleading. We evaluated a practical public health example because it provided experiential risk data that allowed us to assess the practical implications of the published concern that risk matrices would make predictions that are worse than random. We explored this predicted problem by constructing a risk assessment matrix using a public health risk scenario—Tainted blood transfusion infection risk—That provides negative correlation between harm frequency and severity. We estimated the risk from the experiential data and compared these estimates with those provided by the risk assessment matrix. Although we validated the theoretical concern, for these authentic experiential data, the practical scope of the problem was limited. The risk matrix has been widely used in risk assessment. This method should not be abandoned wholesale, but users must address the source of the problem, apply the risk matrix with a full understanding of this problem and use matrix predictions to inform, but not drive decision-making.

## 1. Introduction

Assessing and managing risk is a core element of public health practice, although explicit and detailed documentation of these processes varies among various public health programs. Use of a qualitative (semi-quantitative) risk assessment matrix is a growing practice. The comparative simplicity and apparent ease of use of this approach likely contributes to widespread adoption including a generic international standard for risk assessment techniques in support of risk management [1]. Major public institutions have adopted the risk assessment matrix in fields ranging from assessing highway construction risk, financial risk, preventing terrorist attacks, to agency-wide enterprise risk management across all of government [2,3]. The World Health Organization has adopted this approach for risk assessment of acute public health events [4] and for assuring safe drinking water [5]. Risk matrices have also been adopted nationally in Australia for assuring safe drinking water and for drinking water safety plan implementation in Alberta, Canada [6,7].

Although the various applications of this technique differ in specific details, they all involve the common structural features of a matrix with one axis representing categories of probability (likelihood or frequency) of possible hazardous events and the other axis representing categories of severity (impact or consequences) of those events. Each intersecting cell of the matrix (*i.e.*, row-column pair) is pre-assigned a risk such as low, medium, or high risk. This basic structure is consistent with a widely adopted, if somewhat simplified, concept of risk as being primarily a function of two variables, one representing probability and the other consequences.

The UK National Health Service (NHS) has developed detailed guidance for applying the risk assessment matrix technique, which specified the following properties as being essential for such a risk assessment matrix, “*it should:*

• *be simple to use*;
• *provide consistent results when used by staff from a variety of roles or professions*;
• should be capable of assessing a broad range of risks including clinical, health and safety, financial risks, and reputation; and
• *should be simple for NHS trusts to adapt to meet their specific needs.*” [8]

The ISO standard characterized this technique as offering [1]:

“*Strengths:*

• *relatively easy to use*;
• provides a rapid ranking of risks into different significance levels.

*Limitations:*

• *a matrix should be designed to be appropriate for the circumstances so it may be difficult to have a common system applying across a range of circumstances relevant to an organization*;
• *it is difficult to define the scales unambiguously*;
• *use is very subjective and there tends to be significant variation between raters*;
• *risks cannot be aggregated (i.e.*, *one cannot define that a particular number of low risks or a low risk identified a particular number of times is equivalent to a medium risk)*;
• *it is difficult to combine or compare the level of risk for different categories of consequences*.”

Cox outlined a number of serious deficiencies with the risk assessment matrix approach for assessing risk, including: Poor resolution, ambiguous inputs and outputs, sub-optimal allocation of resources based on inaccurate risk estimation and outright errors in assigning higher rankings to quantitatively lower risks [9]. In particular, for the last concern, Cox demonstrated that the prediction of risk arising from the risk assessment matrix could be worse than a random guess by using a mathematical function for which frequency and severity are negatively correlated and using the commonly adopted formulation (with frequency as a measure of probability and severity as a measure of consequence):

risk = frequency × severity
(1)

Specifically Cox proposed the following theoretical but plausible deterministic negative relationship between frequency and severity values [9]:

frequency = z − severity (for severity between 0 and z)
(2)

He designed a simplified 2 × 2 risk assessment matrix with two categories of frequency (Low, High) and two categories of severity (Low, High), then assigned medium risk to the pairs (frequency, severity) of (Low, High) and (High, Low), high risk to the pair (High, High), and low risk to the pair (Low, Low). He demonstrated that in this risk assessment matrix, most points in the medium risk categories actually have smaller risk values from Equation (1) than any points in the low risk cells. This theoretical example demonstrates that the risk category assignment by the matrix is different from the risk calculation that is intended to accurately estimate the risk and, as such, the risk matrix predictions can be, according to Cox [9], worse than useless (*i.e.*, worse than random).

The prospect of risk predictions being worse than random for risks having a negative correlation between frequency and severity is gravely troubling because such a negative correlation is to be expected in many, if not most, of the circumstances that risk assessment matrix is used to characterize. The wide-spread practice of risk management has reduced the occurrence of hazards causing serious consequences, making their frequency lower. Certainly, for risks being able to accurately distinguish low frequency-high consequence risks from high frequency-low consequence risks is crucial. Despite a growing number of citations, this grave concern of the risk assessment matrix method has received little traction in applied fields such as public health since first proposed by Cox in 2008.

Given our focus on health risk, we sought a practical public health example for which we could find experiential data on risk to assess the practical implications of this concern about risk assessment matrices. Cases, such as drinking water safety, where risk assessment matrices are being widely adopted were not pursued for our analysis because, while there is no shortage of monitoring data, little of this can be readily used for assessing tangible public health risk [10]. The connection between available monitoring data and risk is complex and drinking water disease outbreaks in affluent countries are comparatively rare [11].

The tangible health risks associated with tainted blood transfusions, by comparison, offers a circumstance where, after the major tragedies associated with HIV and hepatitis C transmission through transfusion of tainted blood and blood products, there has been a concerted effort to estimate the frequency of blood contamination for a range of pathogens capable of causing a wide range of disease outcomes of variable severity. Quintela *et al.* produced a generic risk assessment matrix addressing production processes in blood banks, but this analysis did not provide the kind of risk data needed to evaluate the Cox concerns [12].

The objective of our study is to explore the validity of risk matrices for health risk assessment by using a public health risk scenario, tainted blood transfusion infection risk because it provides experiential frequency data estimates for which the frequency of a risk is expected to be negatively correlated with the severity of consequences. That negative correlation is a requirement for allowing risk assessment matrix predictions to be worse than random and potentially harmful according to the analysis of Cox [9].

## 2. Methods

To illustrate the behavior of the risk assessment matrix tool, first we constructed a risk assessment matrix for the hazards associated with infection risk from tainted-blood transfusion using only frequency and severity values. Second, we identified the relationship between frequency and severity values and estimated the risk using Equation (1). Then we compare the estimated risk values (quantitative values) with the risk levels in the risk assessment matrix to verify their compatibility.

**Figure 1:**
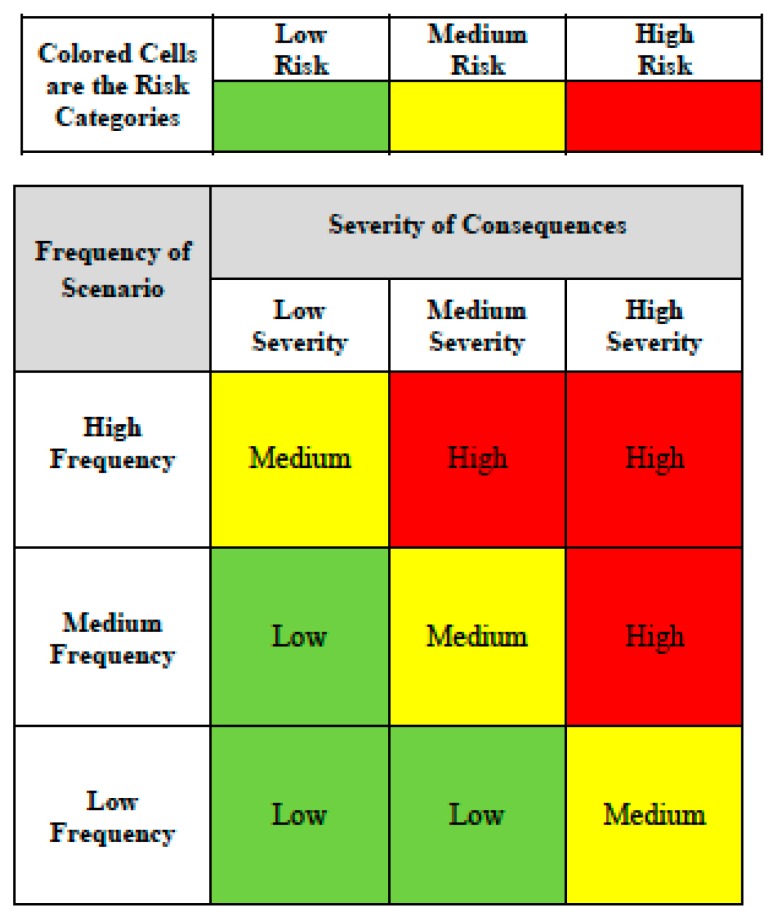
Generic risk assessment matrix.

Risk ranking for decision makers in the risk assessment matrix is commonly visualized by assigning colors to risk categories, which are the cells in the matrix. The assignment of risk categories to the risk assessment matrix (Figure 1) must be done initially by the risk assessor, with an application of judgment, before any specific risks are placed in the matrix. Misunderstanding that this color-coding approach must be restricted to risk has appeared where color-coding was also pre-assigned for both the severity and frequency categories [8]. The color-coding in a risk assessment matrix must only apply to the risk categories that are a product of the severity and frequency ratings that determine the location of any specific risk in the matrix. The magnitude assignment (provided by the color coding) for any risk thus results from its placement in the matrix according to its estimated severity and frequency.

We adapted the NHS criteria for assigning the severity and frequency rankings as listed in Table 1 [8]. To obtain estimates of frequency for our purposes, we collected the prevalence estimates of different blood infectious diseases in blood donors and the population of Canada from the reports of the Public Health Agency of Canada from 1987 to 1996 (Table 2) [13]. For these data we found a very wide range (6 orders of magnitude) of frequency values (0.0000008 to 0.4; Table 2). Because of the wide range of values involved, we adopted a logarithmic scale for both the frequency and severity categories.

Because we located no reports on the prevalence of Creutzfeldt Jakob Disease/variant Creutzfeldt Jakob Disease (CJD/vCJD) in blood donors we used the prevalence in the entire population instead. We acknowledge that this will likely over-estimate the frequency and consequently the risk among blood donors for transmitting CJD/vCJD.

We evaluated the disease severity by assigning severity ranging from very low to very high for each blood infectious disease according to expected complications, mortality, morbidity and available treatment for the infection. While the severity ranking is clearly a judgmental input to the risk assessment matrix based on NHS criteria ranging from very low to very high, frequency is assigned a ranking (extremely low to very high) based on where the frequency evidence dictates (*i.e.*, according to Table 1).

**Table 1:** National health service criteria for severity and frequency levels, adapted from [8].

**Criteria for Severity Levels**	
Very Low Severity	Minimal injury requiring no/minimal intervention or treatment No time off work
Low Severity	Minor injury or illness requiring minor intervention Increase in length of hospital stay by 1–3
Medium Severity	Moderate injury requiring professional intervention Increase in length of hospital stay by 4–15 days Impacts on a small number of patients
High Severity	Major injury leading to long-term incapacity/disability Increase in length of hospital stay by >15 days
Very High Severity	Incidence leading to death Multiple permanent injuries or irreversible health effects Impacts on a large number of patients
**Criteria for Frequency Levels**	
Extremely Low Frequency	Frequency between 0.000001 and 0.0000099
Very Low Frequency	Frequency between 0.00001 and 0.000099
Low Frequency	Frequency between 0.0001 and 0.00099
Medium Frequency	Frequency between 0.001 and 0.0099
High Frequency	Frequency between 0.01 and 0.099
Very High Frequency	Will undoubtedly happen/recur, possibly frequently. Frequency greater than 0.1

**Figure 2:**
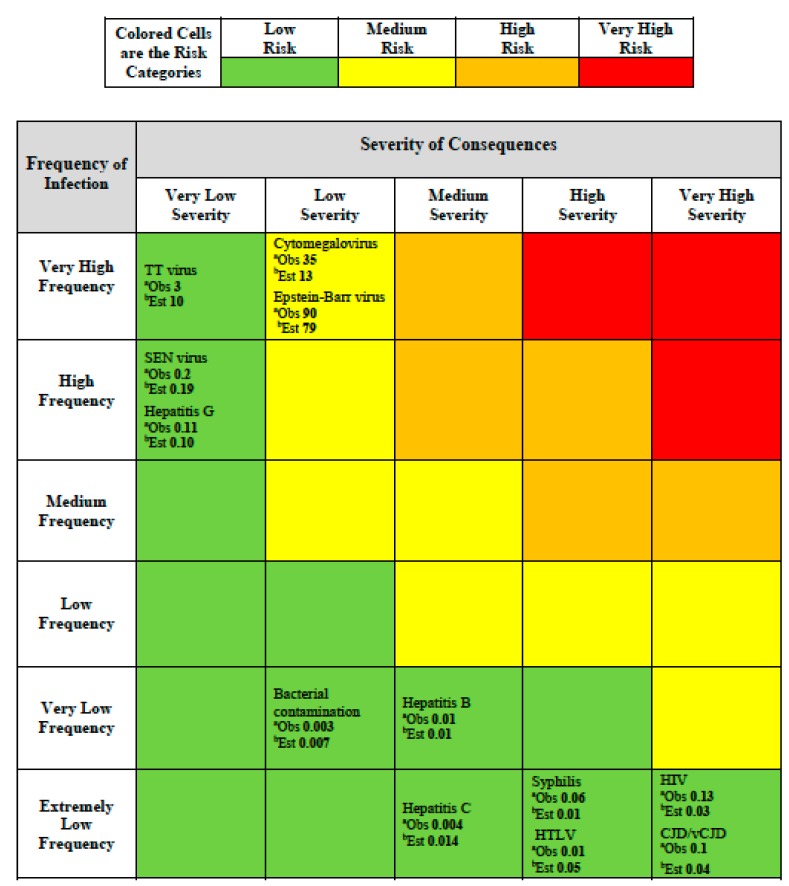
Risk assessment matrix providing colored risk categories plus observed and estimated risk. ^a^ Observed (*Obs*) risk numbers shown are based on the generic risk function (Risk = Frequency × Severity; Equation (1)) and using Table 1 entries for frequency and severity based on Table 2 data; ^b^ Estimated (*Est*) risk numbers shown are based on the fitted risk function Equation (4).

For the matrix scheme we adopted an additional color was added to deal with the wide range of values in frequency and consequences. In our scheme (Figure 2) red indicates very high risk that requires immediate actions and priority in decision-making, orange indicates high risk that requires attention and a control process, yellow indicates moderate risk that requires a specific monitoring program, and green indicates low risk that can be managed according to current standard controls and regulation. The expectation for a risk assessment matrix is that the semi-quantitative ranking provided will be consistent with an underlying quantitative risk ranking which could, at least in theory, be defined by a risk function.

For each infectious hazard in Table 2, we were able to place it in the risk assessment matrix (Figure 2) by considering the frequency and severity category according to the assignments we made in Table 2 according to the NHS scheme (Table 1). In addition, because we have the experience-based estimates of frequency for each hazard and we could use a mid-point of the assigned judgmental severity category from Table 2, we were able to calculate a risk value, using Equation (1). This value is shown for each infectious hazard in Table 2 as the number labeled “*Obs.*” meaning “observed” for each hazard placed in the risk assessment matrix (Figure 2).

**Table 2:** Severity and frequency of blood infectious diseases in Canada, 1987–1996, adapted from [13].

Infectious Diseases	Severity	Severity Category ^a^	Frequency	Frequency Category ^b^	Source
HIV	10^5^	Very High	0.000001	Extremely Low	Blood Donors
HTLV	10^4^	High	0.0000008	Extremely Low	Blood Donors
Hepatitis B	10^3^	Medium	0.00001	Very Low	Blood Donors
Hepatitis C	10^3^	Medium	0.000004	Extremely Low	Blood Donors
Hepatitis G	10	Very Low	0.01	High	Blood Donors
Bacterial Contamination	10^2^	Low	0.000026	Very Low	Blood Donors
Cytomegalovirus	10^2^	Low	0.4	Very High	Blood Donors
Epstein-Barr virus	10^2^	Low	0.9	Very High	Blood Donors
TT virus	10	Very Low	0.3	Very High	Blood Donors
SEN virus	10	Very Low	0.02	High	Blood Donors
CJD/vCJD	10^5^	Very High	0.000001	Extremely Low	Population
Syphilis	10^4^	High	0.000006	Extremely Low	Blood Donors

^a^ Categories assigned using the severity categories provided in Table 1; ^b^ Categories assigned using the frequency categories provided in Table 1.

To allow us to evaluate the concern expressed by Cox, we calculated Spearman’s correlation of frequency and severity in this risk assessment matrix in logarithmic scales to confirm whether the data we were using satisfied the Cox requirement for a negative correlation between severity and frequency [9].

Furthermore, we determined an empirical relationship for log-severity as a function of log-frequency for these infectious disease data, as:

log-Severity = 0.24 log-Frequency^2^ + 1.01 log-Frequency +1.99 (3)

Applying the basic relationship for risk in terms of severity and frequency (Equation (1)) to Equation (3), an empirical equation for risk as a function of frequency can be determined as:

log-Risk = 1.99 + 2.01 log-Frequency + 0.24 log-Frequency^2^ (4)

**Figure 3:**
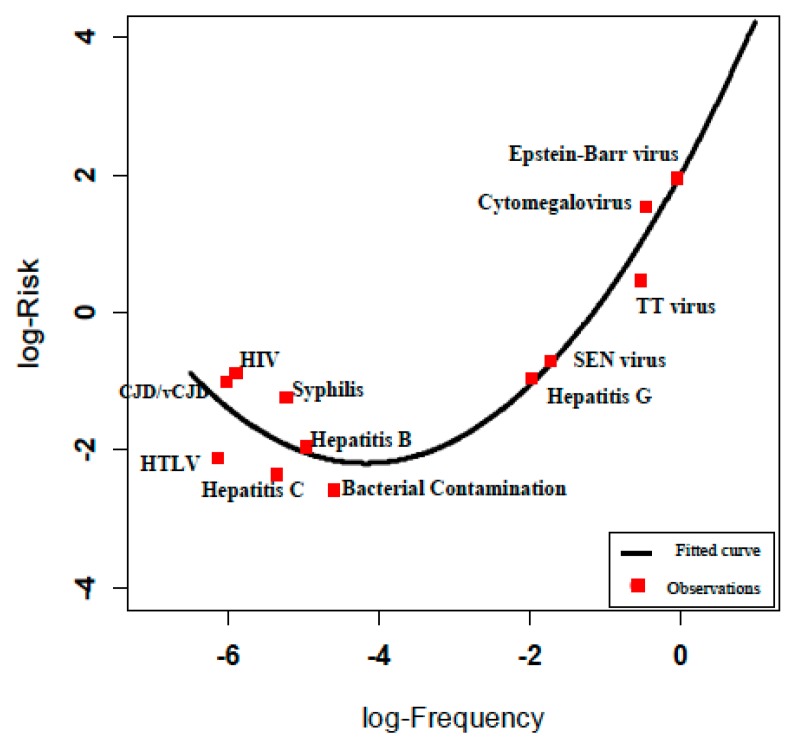
Risk estimation according to log-Risk = log-Frequency + log-Severity.

The relationship between this empirical function and the observed estimates of risk derived from Table 2 is shown in Figure 3.

The calculated risk values for each infection hazard are shown in the risk assessment matrix (Figure 2) for each hazard as “*Est.*” meaning “estimated”. The evidence in Figure 2 does not show any medium, high or very high risks most likely because risk management of blood transfusions has been focused on lowering such extreme risks. However, this lack of higher risk observations challenged our ability to fully assess the concern that Cox raised about the value of predictions raised by risk assessment matrices. Consequently, we attempted to explore this matter further by using the empirical relationship (Equation (4)) we found based on the observed data (Table 2).

**Figure 4:**
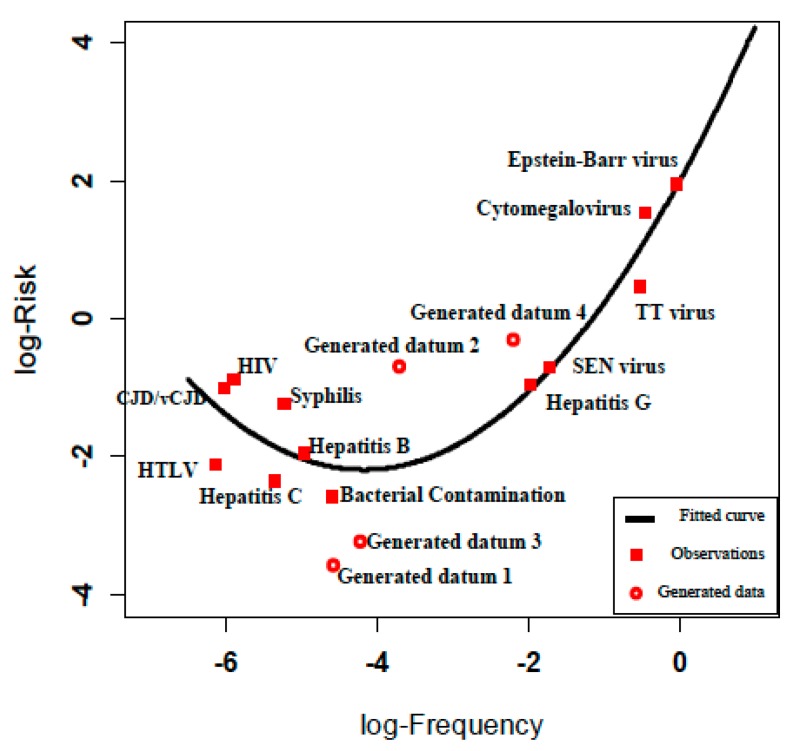
Observed and estimated risk for observations and generated data.

We sought to populate the risk assessment matrix with some generated risk values that were not found in Table 2, but which were consistent with the empirical risk relationship (Equation (4)). For this purpose, we generated four scenarios with frequencies from the prediction interval limits for the new risk estimation in the middle parts (log-frequency between −4.5 and −2), where there are no experiential frequency estimates for blood transfusion infections hazards and calculated their severities accordingly to populate the risk assessment matrix (Figure 4).

We divided the log-frequency gap (−4.5, −2) into three equal parts and selected the two cut points −2.83 and −3.67. The risk estimation for these points using Equation (4) is −1.76 (95% PI: (−3.22, −0.3)) and −2.13 (95% PI: (−3.57, −0.69)), respectively. We generated four data points according to the 95% prediction interval limits of fitted risks. We calculated the corresponding severities from Equation (3) and rounded the values to the nearest severity value (Table 3).

**Figure 5:**
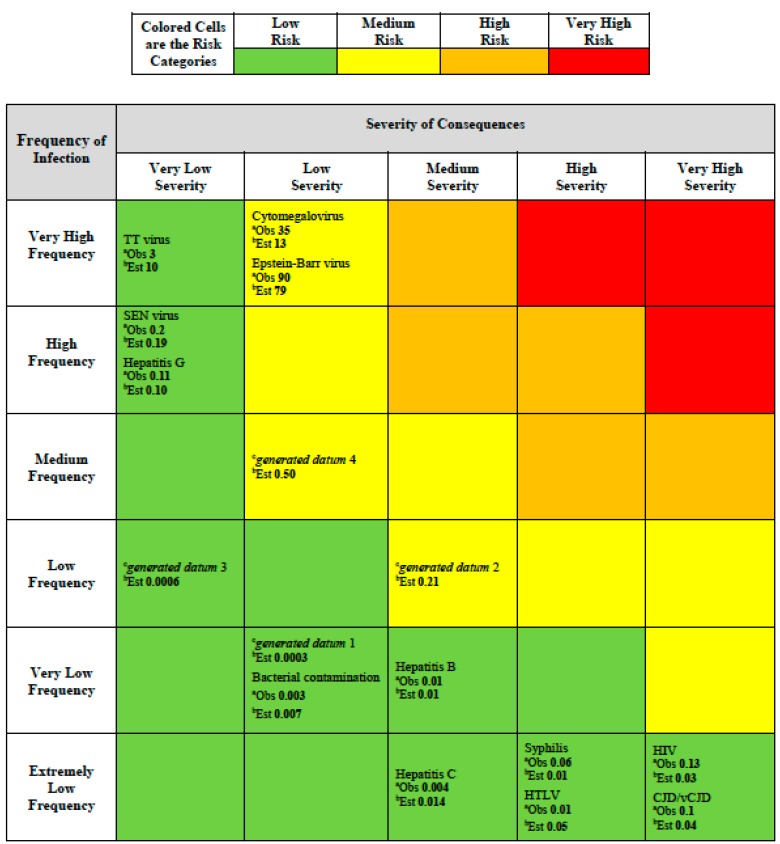
Risk assessment matrix providing colored risk categories plus observed and estimated risk and generated data. ^a^ Observed (*Obs*) risk numbers shown are based on the generic risk function (Risk = Frequency × Severity; Equation (1)) and using Table 1 entries frequency and severity using Table 2 data; ^b^ Estimated (*Est*) risk numbers shown are based on the fitted risk function Equation (4); ^c^ Generated data.

We illustrated the fitted risk curve defined by product of severity and frequency of the diseases (Figure 4). Risks calculated from Equation (4) (reported to 1 significant figure to acknowledge the large uncertainty in these data) are shown on the risk assessment matrix in Figure 5.

**Table 3:** Frequency and severity of generated data.

Generated Data	Frequency	Risk	Severity
Datum 1	0.00003	0.0003	10
Datum 2	0.00021	0.21	1000
Datum 3	0.00006	0.0006	10
Datum 4	0.005	0.5	100

## 3. Results and Discussion

### 3.1. Results

The Spearman correlation between log-severity (S) and log-frequency (F) of blood infectious diseases based on PHAC reports (Table 2) displays a negative correlation of −0.81 which satisfies the theoretical condition prescribed by Cox for creating a fundamental problem with a risk assessment matrix.

The product of this exercise is the risk assessment matrix shown in Figure 2. This is populated according to the blood transfusion hazards provided in Table 2, using the categories proposed by the NHS [8] in Table 1. As expected, given the means used for producing it, the risk assessment matrix apparently distinguishes low and medium risks, *i.e.*, the higher colored risk categories have higher quantitative risks (*i.e.*, the observed values as determined in accordance with Equation (1) for the quantitative values in Table 2). For example, the observed risk value for the Epstein-Barr virus in the medium (yellow) risk category is 90. This estimate is greater than all the observed risk values in the low (green) risk categories, such as TT virus with an observed risk of 3 (Figure 2).

The criticism about range compression for the risk assessment matrix is borne out by finding that the low risk category includes observed risks ranging from 0.003 to 3, a risk range of 1000 fold. In order to test our primary concern, the possibility of the risk assessment matrix making a risk prediction that is worse than random, we had to resort to generating data using the empirical risk relationship (Equation (4)) we found for these hazards.

The four entries in Figure 5, labeled “generated datum” 1 to 4, were calculated to provide us with more data observations in the medium risk category. The generated data points 2 and 4 have estimated risk values of 0.21 and 0.50 and both are categorized in Figure 5 as medium risks. When compared with TT virus, which was categorized as a low risk in Figure 5, we find that it has an estimated (according to Equation (4)) risk of 10. This anomaly illustrates the concern posed by Cox, that the risk assessment matrix provides a risk categorization (color code) that is incorrect in relation to an empirical calculation of the risk [9]. Although we had to resort to generating data from an empirical relationship derived from experiential frequency estimates, we have found that the theoretical concern of Cox can be demonstrated for hazard data derived from authentic experience.

### 3.2. Discussion

Given the wide-spread and apparently growing popularity of risk matrices for risk assessment purposes, the prospect of obtaining results that are worse than random is clearly a serious concern. Yet, we have found little practical uptake of Cox’s concerns evident in public health relevant literature in the six years since being published. Wieland *et al.* referred to the Cox critique of risk assessment matrices in relation to the limited resolution of the method possibly leading to an overestimation of risk for an evaluation of qualitative risk assessment of the spread of African Swine fever [14]. Pickering and Cowley provide an extensive critique of risk assessment matrices, including citing criticisms by Cox but they do not address the specific concern about risk assessment matrices being worse than random for cases in which there is a negative correlation between frequency and severity of risk [15]. Hubbard and Evans present a number of arguments against all common judgmental scoring methods for risk assessment, including the steps necessary to construct risk assessment matrices, but they only refer to Cox with respect to range compression and loss of resolution [16]. Holt *et al.* took note of the limitations of risk assessment matrix structure in their review of tools for guiding decisions in relation to assessing risks from pests [17].

Levine referred to Cox in criticizing risk assessment matrices for failing to acknowledge uncertainty in the rating of risks according to the axes categories, ignoring information on how to best manage risks or to acknowledge the decision-maker’s risk preferences [18]. Levine’s main concern was also the range compression, which he proposed to remedy by using logarithmic scales to reflect the large range of values that often exist. Regarding the flaw that Cox has described, Levine only concluded without elaboration: “*When used to assess a set of hazards with a negative correlation between frequency and consequence, risk matrices are often uninformative and occasionally misleading*.”

Ball and Watt have provided the most complete evaluation of the practical problems with risk assessment matrices [19]. They acknowledge the potential for erroneous risk ranking described by Cox but go further after they observe that he: “*Determines that risk matrices are limited in their ability to rank risks correctly and further that they should not be used as they often are, that is, as proxies for risk management decisions by the simple device of overlaying them with colors associated with risk management priorities. This is because optimal resource allocation is quite obviously a function of far more than the two dimensions of likelihood and consequence upon which the matrix rests*.” Their valid concerns about over-simplification of risk are elaborated by richer, more comprehensive definitions of risk than provided by Equation (1) (probability × consequences), which acknowledge the inherently multidimensional character of risk and the inevitable reliance of risk assessment on subjective estimates [20,21,22].

In practice, the risk assessment matrix is constructed based on possible hazards, but without any prior assumption on relationship between frequency of hazard and its severity. Our illustration with a tangible public health risk scenario provides insight into limitations of the risk assessment matrix for guiding decision making for the common circumstance where the frequency of hazard and its consequence are negatively correlated. Decision makers need to identify the expected correlation between frequency and severity and recognize that where a negative correlation exists, the risk assessment matrix categorization of risk might not reflect the quantitative risk estimates in accordance with an assumed risk function and may well mislead decision-makers with a worse than random assessment of risk [9].

A tangible, pragmatic approach to the Cox problem for risk assessment matrices has been illustrated in a risk management approach to support the implementation of drinking water safety plans in Alberta, Canada [7]. In this approach, the rating scheme assigns numerical scores for frequency and consequence as well as the generic risk function (risk = frequency × consequence) that thereby defines where in the risk assessment matrix evaluated risks will be plotted. This predefined approach for constructing the risk matrix relies on the validity of the predetermined assigned numerical ratings, but it likely avoids the problems of creating predictions that are worse than random. Of course, all the other practical limitations and associated cautions for the risk assessment matrix that have been summarized earlier remain valid concerns for such simplified applications.

## 4. Conclusions

Our limited validation of the Cox concern, using a tangible public health risk example, suggests a need for careful reconsideration of uses of the risk assessment matrix in risk management. There is no straightforward solution to address the concerns raised about risk assessment matrices. We do not propose a viable alternative to the risk assessment matrix tool for mapping risks that lack prior knowledge on harm frequency and its severity. However, risk analysts in all fields using the risk assessment matrix should be aware of this limitation. At least, they should investigate or contemplate the plausible correlation between frequency and severity for the hazards to be evaluated in the risk assessment matrix according to their prior knowledge in the field. When some data are available (generally not the case), they could look at data in the manner we did and try to fit a risk function and eventually compare the results with the risk assessment matrix results to identify anomalies.

We do not advocate a wholesale abandonment of risk assessment matrices for guiding risk management, particularly when applied, as they commonly are, to diverse hazards across a broad organizational portfolio. Of course, application of the risk matrix to a diverse range of hazards brings its own complications and challenges that must be acknowledged. The construction and evaluation of a risk assessment matrix can, if used wisely, stimulate a valuable discussion among operational personnel to reflect on what can go wrong and how well prepared the organization is equipped to manage various risks. Provided that the results of a risk assessment matrix exercise are treated with appropriate and healthy scepticism, they can serve a useful purpose for initiating and focusing a discussion about risk priorities within an organization. Achieving healthy scepticism may be difficult as long as risk matrix users see this technique as a simple tool and ignore the embedded complexity involved.

The primary danger revealed in this analysis, owing largely to the pioneering insight offered by Cox [9], is to avoid allowing such over-simplified risk analyses to become the risk management decision rather than properly being only an operational input that can guide, challenge and inform decision-making to be based on a comprehensive understanding of risk. Risk assessment matrix outputs should not be allowed primarily to drive or, in the worst case, to become the risk management decision.
